# Patent cliff and strategic switch: exploring strategic design possibilities in the pharmaceutical industry

**DOI:** 10.1186/s40064-016-2323-1

**Published:** 2016-05-23

**Authors:** Chie Hoon Song, Jeung-Whan Han

**Affiliations:** Research Center for Epigenome Regulation, School of Pharmacy, Sungkyunkwan University, Suwon, Republic of Korea

**Keywords:** Patent expiration, Decision making, Strategic management, Strategy, Pharmaceutical industry

## Abstract

Extending the period of the market exclusivity and responding properly to the recent agglomeration of patent expiries are pivotal to the success of pharmaceutical companies. Declining R&D productivity, rising costs of commercialization, near-term patent expirations for many top-selling drugs are forcing companies to adopt new systems to introduce innovative products to market and to focus on strategies that increase the returns from the existing product portfolio. This systematic review explores various strategic and tactical management approaches by synthesizing the relevant literature and practical examples on patent expiration strategies. It further discusses how the mix of competition policies and strategic instruments can be used to maintain declining revenue streams from the blockbuster business model of the pharmaceutical industry. The review provides a comprehensive overview of the research on various strategies, offers both theoretical and practical guidelines for strategy transformation that companies can use to prolong the market exclusivity, and identifies knowledge gaps that needed to be addressed in order to improve efficiency in policy design.

## Background

**Fig. 1:**
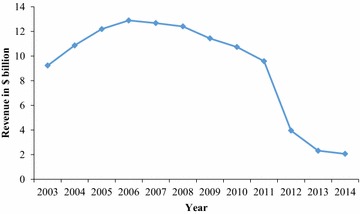
Worldwide revenue profiles of Pfizer’s Lipitor^®^ 2003–2014 (*Source*: annual and financial report from Pfizer)

Ensuring the long-term profitability and revenue upon launching of new drugs raises serious concerns for the involved parties in the pharmaceutical industry. In many industries, the loss of patent-protected exclusivity is known to be followed by severe losses in sales and profit to incumbent companies. When the patent protection expires, generic manufacturers enter the market with drugs that are equivalent to the innovator’s drug, but typically at a significantly lower price (Pearce 2006). The investigation carried out by European Commission has shown that the average generic price 2 years after its entry is around 40 % below the price of the former brand name products (European Commission 2009). This helps to ease pressure on public health budgets, contributes to increased consumer welfare and creates incentives for future research, but the innovator-company^1^ is confronted with challenges as a result of drastically falling revenues. The sales dynamics of Pfizer’s Lipitor^®^, formerly known as the best-selling prescription drug in history, show a dramatic decrease in sales, once the period of market exclusivity is exhausted in 2012 (Fig. 1).

Against this background, a dependence on patent-protected assets means that the pharmaceutical industry relies on repeated R&D successes, whereby the risk is not evenly spread throughout the sector.

The immediate decline in revenue after the patent expiration is referred to as “patent cliff” (Jimenez 2012). Patent cliffs are one of the major issues facing the pharmaceutical industry, as the major blockbuster drugs (i.e. the annual global revenue greater than $1 billion) typically account for a large percentage of company turnovers. A number of blockbuster drugs have lost their patent protection in recent years and more will do so in upcoming years. The loss of exclusivity with regard to one or more important products can have a significant negative impact on the company’s performance and is forcing “Big Pharma” to look for new revenue streams. There is no doubt that patent expirations triggered some of the dramatic merger and acquisition announcements that have occurred over the past years (Mittra 2007). A number of selected major patent expiries are given in Table 1. It becomes evident that several pharmaceutical companies will be confronted with another big wave of patent expiration within next years.

**Table 1:** Patent expiry of blockbuster drug in 2016 [*Source*: IMS (2012)]

Drug (trade name)	Date of expiration/country	Company	Application area
Alimta^®^	2016/US	Eli Lilly	Cancer
Benicar^®^	2016/US	Daiichi Sankyo	Blood pressure medication
Benicar HTC^®^	2016/US	Daiichi Sankyo	Blood pressure medication
Crestor^®^	2016/US	Astra Zeneca	Lowering LDL cholesterol
Cubicin^®^	2016/US	Merck	Bacterial infection
Zetia^®^	2016/US	Merck	Lowering LDL cholesterol
Baraclude^®^	2016/JP	Bristol–Myers Squibb	Antiviral medication (hepatitis B virus)
Glivec^®^	2016/EU	Novartis	Myeloid leukemia
Vfend^®^	2016/EU	Pfizer	Antifungal medication

Patent expirations dates are subject to change due to patent litigation, additional patents and other market exclusivities. Patent expirations do not imply immediate generic availability

Consequently, the current “blockbuster business model” is designed in a manner that the innovator-company is guaranteed to lose the core business portfolio every 10–12 years after market entry. The impending patent cliff is set to erode more than $100 billion in sales for drugs going off-patent in the coming years, leaving a financial vacuum for affected companies (Denoon and Vollebregt 2010). While the first wave of patent cliff left deep marks on the operating results and financial position, pharmaceutical companies now seem to be better prepared to face the upcoming second wave of patent cliff. But still, as it becomes increasingly important to defend revenues from an existing product portfolio and slow down the sales losses associated with the market entry of generics, there is a need to better understand and critically review the strategic options for research-driven pharmaceutical companies. Although, there is no one-size-fits-all solution to entirely prevent the decline in revenue, the overall financial picture on choosing the adequate and carefully implemented strategy will turn out to be more favorable than that of unwisely implemented strategy. In view of these concerns, this paper attempts to address the ongoing challenges of patent cliff at the interface of price-based competition and innovation policy. Various strategic aspects are brought together, reviewed and synthesized in order to derive meaningful conclusions and options for action based on this approach. A broad range of literature was assessed to answer a set of questions related to strategic choices and how other strand of innovation research may be integrated to sustain the competitiveness of branded product beyond the end of market exclusivity. The resulting framework aims at facilitating the decision-making process regarding the identification of appropriate strategies and consensus-building in the organization.

The paper is organized as follows: in “The pharma industry: an interesting avenue for designing strategies” section, a brief background on the business landscape of the pharmaceutical industries is provided, and the driving forces behind patent cliff and the impact of generic entry for innovator-companies are discussed. In “Strategic choices in the case of patent cliff” section, several strategic options to mitigate the impact of patent cliff are summarized and critically reflected. Reflecting on these findings, the paper offers insight into how these interrelated strategic building blocks interact with each other to create a basis for strategic realignment. “Conclusions” section provides summary and concluding remarks.

## The pharma industry: an interesting avenue for designing strategies

The pharmaceutical industry has a unique approach to its research and development compared to other industry segment. The sector is strongly R&D driven, highly regulated and is characterized by an increasing level of product complexity and quality requirements (Grabowski 2004). It generally takes up more than 10 years and at least $1 billion to successfully develop and achieve a market approval of a new blockbuster drug (efpia 2014). The long development and testing cycles together with uncertain prospects for commercial success make it demanding to delay planning and decision making processes within an organization.

Over the years, the pharmaceutical industry has constantly adapted its business model towards the development of a single drug that targets a broader population. This approach has contributed to important advances in pharmacology to treat a wide spectrum of diseases while ignoring the patient’s individual biology. However, as many companies are conducting researches in similar indication areas and working on influencing the same enzyme activity or the interaction with receptors, it seems opportune to file a patent application as early as possible for the discovered drug candidate. This approach has the disadvantage that the patent expiration is expected to occur much earlier than usual and the effective market life of drugs is significantly reduced (Hemphill and Sampat 2012). It takes an average of 12–13 years to complete the research and development activities, from the initial patent filing to the regulatory approval of new drug, thereby reducing the effective time of market exclusivity to 7–8 years (efpia 2014). Grabowski and Moe (2008) emphasized that the shortened exclusivity period offers “insufficient time for most new drugs to recoup the up-front R&D costs and earn a positive return on this investment”. Subsequently, reducing the time necessary to develop and commercialize the product is one of the key success parameters. However, as the innovator-companies generally have a comparatively limited portfolio of innovative products in their pipeline, they can “no longer simply allow post-patent profits to be eroded and rely on new, patented products to replace their lost revenues” (Bruce 2003). In this context, strategic behavior can be expected in a legal framework to promote lifecycle extension strategies. A steady communication between the health care providers and representative of the pharmaceutical industry is seen as an essential element in making the health care more affordable for the payers and profitable for the industry. Thus, the patent cliff might provide a unique opportunity for the participant in the current healthcare system to collaborate and reinvent the current model of drug discovery and drug marketing for the sustainable development of the whole industry sector.

Consequently, a considerable amount of efforts has been directed towards elaborating strategies for dealing with diversity. Despite the high organizational and strategic expenditure, there is still a widespread uncertainty as to what degree the implemented strategies can contribute to coping with the complexity. It is considered that the subsequent decline in revenue upon patent expiry is inevitable, but the introduction of new business logic and an analytical approach to value capturing will lead to higher efficiency levels of certain process steps.

### Factors driving the patent cliff

There are several reasons for generic entry and the associated patent cliff. Traditionally, the innovator-companies have attempted to replace the blockbusters with new drugs from their pipeline to offset the looming decline in sales. However, the R&D productivity of the pharmaceutical industry has declined continuously over the last decade accompanied by increasing costs of bringing new drug to market and delaying of drug approval processes by government agencies (Pammolli et al. 2011). Such maturity problems result from the fact that the companies have struggled to develop and market products that are effective enough to compete with existing product ranges and meet regulatory requirements (Mittra and Tait 2012). The shrinking pipeline of research-driven pharmaceutical companies and the generic competition have caused them to deploy a range of strategic approaches (Paul et al. 2010), such as the acquisition of biotech companies to refill their R&D pipelines (James 2002). For instance, Pfizer acquired Wyeth Pharmaceuticals in 2009 for $68 billion upon nearing the patent expiry of Lipitor and not having a proper replacement in company’s innovation pipeline (Malik 2009). The rationale behind such acquisition is to enhance the earnings performance in the cliff period, to increase breadth and depth of its portfolio into biologics and vaccines and to diversify its source of revenue by investing in complementary businesses.

The Hatch–Waxman Act, also known as the Drug Price Competition and Patent Term Restoration Act, in 1984 have changed the dynamics of generic entry on branded drugs. It intended to expedite generic drug approval and to encourage generic participation in the prescription drug industry and has positively contributed to the creation of modern generic drug industry in US (Grabowski and Vernon 2000). Title I of this legislation established the Abbreviated New Drug Application (ANDA) process, which grants a 180-days market exclusivity period to the first generic manufacturers who successfully files an ANDA. Under this legislation, a manufacturer only needs to demonstrate the bioequivalence of the drug. Before its adoption, there has been no streamlined drug approval process for generics and generic suppliers were obliged to go through the same costly and time-consuming clinical trials to obtain a market approval. This incentive has driven the generic competition, promoting the prompt entry of generics to the market. Moreover, the number of generic entrants is determined by pre-expiration brand revenue, length of market exclusivity period and the ease of manufacturing (Scott Morton 2000; Grabowski and Kyle 2007). Naturally higher sales and shorter market exclusivity periods for the branded drug attract more generic entry. Additionally, the gradual decrease in market share can be linked with branded drug withdrawing the pre-expiration brand advertising.

The evolving payer initiatives to reward generic use have also led to the erosion of branded drugs. Payers are becoming more effective in their efforts to influence the way prescriptions are issued and dispensed by pharmacists (Tuttle et al. 2004). In response to rising healthcare spending, governments in developed economies are supportive towards generic production to keep costs under control and to make public health system sustainable. For instance, there exist policies supporting the sale of generic medicines by obliging pharmacists to always dispense the cheapest product (European Commission 2009). This may lead to a higher generic share. In addition, the increasing number of antitrust investigations and monitoring of patent settlements between pharmaceutical companies that might violate the competition law put pressure on innovator-company to refrain from applying generic-entry-delaying, anti-competitive practices such as reaching the pay-for-delay agreements (OECD 2014). Growing pressure from the healthcare regulatory authorities and the growth of managed care organizations have intensified price competition significantly in the United States and in varying degrees in some other countries. Shortcomings in the regulatory framework, which does not favor streamlined trials for scrutinizing added value of novel medicine and the increasing efficiency of generic competition in launching and marketing of new drugs, are the dynamic force behind the patent cliff.

In addition, a greater understanding in the molecular basis of human disease and pharmacogenomics has led to the emergence of therapeutic interventions tailor-made for stratified group of patients. Such medicines are likely to improve clinical outcomes and increase patient safety, which the current blockbuster drug struggles to deliver. Drugs that target at smaller patient population have already achieved the market breakthrough. Such transformation would require a fundamental restructuring and re-tooling in the drug discovery and development.

### Impact of generic entry for the innovator-company

Generic entry has consequences for many different market players. It leads to substantial changes in the average price of drug, thereby shifting the competition focus from monopoly towards competition based on price. Therefore, innovator-companies are forced to implement profit-raising measures by either adapting the prices of pharmaceuticals sold under its brand name or finding new source of profit, ideally in the form of new blockbuster drugs. Eli Lilly’s anti-depressant Prozac^®^ lost about 70 % of its market share within the first 20 weeks of the market entry of its replica (Druss et al. 2004). Such rapid revenue loss for innovator-company calls for the incorporation of sophisticated product lifecycle management, which allows for maximizing a product’s lifetime value through optimal use of patent system (Prajapati et al. 2013).

Two major events have emerged over the course of the years to respond to this new economic environment. Firstly, patent cliff induced the pharmaceutical industry into a new wave of product innovation. Competition by generics can be regarded as a dynamic force, which stimulates pharmaceutical companies to continue to invest in the research and development of innovative treatments (European Commission 2009). Manufacturers of generic products typically invest significantly less in R&D than the innovator-company and are accordingly able to price their products significantly lower than branded product. In the pharmaceutical industry, the end of patent life is a part of the life cycle. The “low hanging fruits” in drug development have been picked to a large extent (Williams 2011) and it is necessary to protect their revenue stream by measures other than unjustifiably abusing the intellectual property right at the expense of competition and public welfare (Glasgow 2001). Therefore, pharmaceutical companies are encouraged to focus on specialty drugs with low substitution potential by creating the so-called “niche busters” (Dolgin 2010; Kakkar and Dahiya 2014). By focusing on niche market, companies can concentrate on their core competencies, thus freeing up the resources and getting rid of assets that have less importance for competing on a global scale. One example is the agreement between Novartis and GlaxoSmithKline (GSK), whereby Novartis purchased the oncology business from GSK and GSK obtained the vaccine business in exchange.

Secondly, a substantial effort has been put into developing new business model and evaluating the legislative practice that suit the changed business environment (Rusu et al. 2011). While the business environment has changed significantly, the respective business model has not kept pace. If revenues can only be sustained by exploiting the legislative loopholes, companies willing to invest in the development of medicine will face a serious dilemma on a long-term scale. Without proper policy intervention, the rise in patent challenges will not only shorten the effective market life, but also contribute to the dearth of high-risk and high-necessity drugs (Higgins and Graham 2009). The predominant version of current business model, at which an extensive marketing effort and sales presence are crucial for maintaining streams of revenue, will be less effective. In future, most medicines will likely be paid for on the basis of the results they deliver and pharmaceutical companies need to pursue the path of “profiting together” instead of “profiting alone” by moving into health management space and to go beyond medicine (pwc 2009).

With rising R&D expenditure, it is important for pharmaceutical companies to make positive returns from the innovation. This can be only achieved by improving both effectiveness and efficiency. The use of generics is expected to continue in the future, as the government promotes policies to use low-priced alternatives. Thus, innovator-companies need to combine various instruments from the strategic tool box, which will be presented in the following, to achieve a balance between innovation and reward.

## Strategic choices in the case of patent cliff

**Fig. 2:**
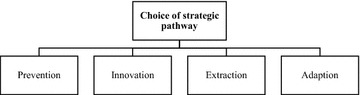
Overview of the four generic strategic pathways [*Source*: modelled after Raasch (2006)]

Research-driven pharmaceutical companies can employ a range of strategies to extend their existing patent protection on therapeutically active substances or pharmaceutical composition, thus maximizing the commercial value of the product and retaining its market share. Four generic strategic pathways exist, which can be considered as a point of departure for companies in outlining and devising their own strategies. Differing levels of strategic and marketing dimension define different landscape for action. For instance, considering the marketing mix from the 4P perspective (product, price, place and promotion), the variable “place”, which determines the intensity and manner how products will be made available (van Waterschoot and Van den Bulte 1992), plays a minor role in the marketing of pharmaceuticals, since the prescription drug market is regulated by state authorities and there are hardly any entrepreneurial flexibility with regard to the distribution channel. Additionally, depending on the time period, a different mix of strategies is required to effectively prolong the life cycle of the drug and to balance risk and reward. In simplified terms, the strategic decisions regarding the variable “product” need to be taken at an early stage, as the development of product innovation usually takes time, while “promotion” and “price” can be adapted more flexible to the requirements and ambient conditions on short notice. Thus, determining which pathway to pursue depends largely on company’s existing capabilities, opportunities and priorities. Figure 2 gives an overview of the four generic strategic pathways.

In the following, the strategic options available to pharmaceutical companies confronted with patent cliff are classified into above categorization and the resulting implications are discussed.

### Prevention strategy

The first generic strategic orientation to extend the patent protection is to temporarily prevent or distort competition. The basic principle of ‘prevention strategy’ is therefore to exploit possibilities for extension of market exclusivity mostly by means of legal measures. One commonly applied strategy is related to strategic patenting. Patents are the primary tools that the research-driven companies use to establish and maintain their brands in the marketplace and provide a window of opportunity to enforce the exclusivity of the inventions (Cantrell 2009). Pharmaceutical industry has adopted a strategy of filing multiple patents to protect its branded drug. This practice of forming a network surrounding the base patent, is called creating “patent clusters”.^2^ The acquisition of secondary patents, obtaining features other than the main active drug ingredient (such as crystalline forms of the original compound, methods of use or formulations), can create a solid portfolio covering different aspects of the drug (Burdon and Sloper 2003). For instance, if the manufacturing process is optimized after filing a patent application so that the new synthesis method did not have to be disclosed at the time of initial filing, the related process patent, such as enhancing of purity level, can be filed at a later stage of the product lifecycle. Additionally, the primary patent may be split into several patents. One patent may seek protection for a broad genus encompassing various compounds, while another patent may comprise a claim related to a specific compound. In some cases, if one isomer^3^ is found to be more active than the other or offers substantial and previously unpredicted therapeutic advantages over others, it may provide a basis for a separate patenting for the more beneficial isomer (Hutchins 2003). Accordingly, secondary patents encompass inventions directed to the incremental improvement of the primary patent and would permit the innovator-company to maintain the market share, even if the generic producers try to enter the market by contesting the validity of the primary patent. Amin and Kesselheim (2012) reported that a large cluster of secondary patents related to HIV medication (ritonavir) could delay generic competition 12 years after the expiration of the patents on the drug’s base compound. The Sector Inquiry by European Commission has revealed that there is a trend for companies to continuously file patent applications as the expiry date of the primary patent approaches, whereby the ratio of primary to secondary patents is 1:7. This kind of strategic patenting intends to build portfolios of patents for a defensive rather than for inventive purposes, placing the innovator-company in a more favorable position for the patent-related disputes concerning the launch of generics. Glasgow (2001) concludes: “[…] intellectual property protection is not being used to promote an incentive to create and innovate. Rather, intellectual property rights are being used to gain and maintain an exclusive market share for the most profitable, not necessarily the most beneficial, drug”. Consequently, the term “evergreening” indicates the strategic maneuver to intentionally extend the market monopoly beyond the known patent life through secondary patenting (Dwivedi et al. 2010). The consequent patent maze from secondary patents can result in difficulties for generic suppliers to determine when relevant patents will expire and when it is safe to enter the market without inadvertently running into patent infringement problems. Even if the generic suppliers have success in maintaining a clear view through the multiple layers of patent protection, they find themselves at a disadvantage in that they might be prohibited from using the compound produced by the most economical route or using the most stable forms of the drug (Hutchins 2003). However, more recently, patent-related legal challenges from generic suppliers have been more successful and the lead time for the market launch of generic products has become much shorter (European Commission 2009). The secondary patents may not cover the proposed generic product properly and are contestable (Glass 2004). At the same time, it is important to keep in mind that there is a tendency to restrict the patentability of secondary patents, especially in developing countries, leading to questionable patents on highly prolific medication to receive a strong second look. In brief, strategic patenting behavior can deter generic entry, as the costs to invent around or challenge the patent maze can be detrimental, but stricter patentability standards make the strategic patenting more vulnerable.

Another possibility to extend the market exclusivity by pursuing legal avenues is provided through obtaining of supplementary protection certificates (SPCs). SPCs are an additional protective mechanism introduced by EU to serve as an extension to the patent right (Hitchcock and Tugal 2003). For the pharmaceutical sector, SPCs can be issued to compensate the efforts put into research and development and the elapsed period between the patent filing and obtaining market authorization to place the approved drug on the market. SPCs extend the effective protection of products already on the market by a maximum of 5 years upon patent expiry. However, the protection granted through SPC can be legally challenged and declared as invalid. A similar practice has been adopted by US and Japan under the name of “patent term restoration” in the 1980s. In US, the innovator-companies can apply for up to five additional years of patent protection for the new drug to make up for the time lost while the product was subject to the FDA’s regulatory review (Title II of the Hatch–Waxman Act) (Agrawal and Thakkar 1997). Building on this legislation, brand owners can file a patent infringement suit, after an ANDA with paragraph IV certification is filed by a generic manufacturer.^4^ The FDA cannot approve the ANDA until the court decision, thereby leading up to 30 months extension of market exclusivity (Bhat 2005).

Another way of extending the market exclusivity is to apply for orphan drug status. In EU, orphan drug status is granted to drugs for treating rare (life-threatening or chronically debilitating) diseases affecting not more than 5 in 10,000 for which there is currently no adequate or possible treatment, while making them sufficiently profitable to bring to market (EMA 2015). Historically, a rare disease is not addressed by the pharmaceutical industry because of its small number of patients. The regulation encompassing the orphan drug designation in EU, which was laid down in early 2000, grants 10 years of market exclusivity, acceleration of the authorization procedure and reduction of administration fees (Haffner et al. 2008). In some cases, an already authorized medicinal product may seek accreditation for an as orphan drug designated indication. Due to the expanded range of application as well as the possibility to use the new indication as a distinguishing feature with respect to the generic competitors, the turnover of the patent-free product can be maintained. However, attaining the orphan-drug-status cannot completely prevent the generic manufacturers from competing in the same market segment, since the prescriber can administer the generics in the category of “off-label” use. In contrast, the Orphan Drug Act has been in force since 1983 in US. The law provides several similar economic and regulatory incentives, including 7 years of market exclusivity, fast-track approval and tax credit, to encourage the development of orphan drugs (Minghetti et al. 2000). However, it has been suggested that orphan drug regulations have not quite lived up to their expectations, thus the current regulations should be revised to provide improved incentives for industry and for better priority-setting of development (Tambuyzer 2010). Besides, companies can be granted a 6-month patent term extension for submitting pediatric clinical trials (Kvesic 2008).

Generic entrants might challenge the validity of the patents enjoyed by the brand owner, either by entering the market at risk or taking the case to the court. In this context, the brand owner and generic companies are entitled to reach an out-of-court agreement and settle their patent litigation to avoid the costly expenses of pursuing legal action at court. In order to prevent generic entry, the brand owner agrees with generic competitors to not enter the market in return for substantial payment or conveniences, granting the equivalent of what they would have earned upon the market entry (Bulow 2004). This strategy is only applicable if the expected profit from the market dominance exceeds the payments. The generic competitors would stay out of the market for the duration of the agreements and the brand owner can enjoy an effect similar to successfully enforcing its patent right. However, out-of-court agreements between companies in relation to patent litigation are not immune from competition law scrutiny (OECD 2014). Although companies have a legitimate interest in finding a mutually acceptable compromise through settlements, some form of settlements may be problematic from a competition law perspective. In recent years, the anticompetitive practices such as the pay-for-delay agreements in the context of patent settlement are under intensified monitoring of competition authorities and should be coordinated with care, since they can entail the risk of high penalty payments and damage to company’s reputation and are detrimental to the interest of the communities (Table 2).

**Table 2:** Overview of prevention strategies

Strategic options	Description	Exclusivity period	Source
Strategic patenting (“later issued patents”)	Obtaining patent protection on different aspects around the base compound patent	20 years from the date of filing	Hutchins (2003), Burdon and Sloper (2003)
Patent term restoration	Granting of additional market exclusivity for the time lost due to FDA approval process (Title II of Hatch–Waxman Act)	Maximum of 5 years	Agrawal and Thakkar (1997)
SPC	Protective mechanism serving as an extension to patent right	Maximum of 5 years	Hitchcock and Tugal (2003)
30-month stay provision	Filing a patent infringement suit to fight ANDA	30 months from the date of notice or till court decision	Bhat (2005)
Orphan drug	Applying for orphan drug status for an already authorized drug	7 years of market exclusivity in US, 10 years in EU	Haffner et al. (2008), Minghetti et al. (2000)
Pediatric exclusivity	Submission of pediatric clinical trials on the FDA’s request	6 months of market exclusivity	Kvesic (2008)
Patent settlement agreements	Involving in settlements with generic manufacturers to delay the market entry	Duration of the agreement	Bulow (2004)

### Innovation strategy

The second generic strategic orientation is to avoid competition through product or business model innovation. The basic principle of ‘innovation strategy’ is therefore to outpace the competition with a new move on the perceived value of a product or business model. Typically, the ability to continually innovate and actively provoking shifts in industry evolution are the hallmark of a successful outpacing strategy (Gilbert and Strebel 1987). In this sense, product-related innovation strategies comprise product-line extensions, approval in new indications, introduction of follow-on product and Rx-to-OTC-Switch, which all build on company’s knowledge assets and assets complementary to them (e.g. structural assets).

Product-line extensions refer to a variation of the existing products by proactive measures aimed at advancing it. This implies that either the drug itself is altered or the product manufacturing procedure is improved to achieve a higher level of purity or to achieve cost saving potential (Dubey and Dubey 2009). In the first case, superior formulations of the known compound can promote patient compliance through reduced dosing, new route of administration or improved compatibility (less side effects). After the patent expiration of the blockbuster drug Prozac^®^ (fluoxetine) in 2001, Eli Lilly obtained patent protection for the combined use of olanzapine and fluoxetine (Symbyax^®^) for treatment of refractory depression. The patent of this new combination drug ends in 2017. This kind of product bundling approach is a popular mechanism to prolong the product lifecycle, since the bundling can facilitate the leveraging of market power from one to another. Pfizer’s Procardia XL^®^ is a result of line extension with controlled-release formulation for the treatment of arterial hypertension. Such reformulations build on same active ingredient of the original drug and have a shorter approval path. Hong et al. (2005) reported that a product-line extension helps to maintain the price level to a reasonable extent despite the entry of generic suppliers. Moreover, the shift from racemic mixture, compound consisting of an equal mixture of a pair of enantiomers, to a single isomer can entail potential for an improved drug efficacy or less complex pharmacokinetic and pharmacodynamic profile. One example is Prilosec^®^ (omeprazole) from AstraZeneca. The company has successfully marketed Nexium^®^, which is the single (S)-enantiomer of omeprazole, as an extension.

Indication extension refers to the practice of identifying novel ways of application for an existing drug. In the course of clinical use, effects and accompanying symptoms of the drug, which were not manifested during the clinical study and not familiar at the time of registration, become clear. If the resulting evidence on possible new indications appears to be promising, an extension of the drug approval may be requested. Upon receiving approval for reformulation or new indications, innovator-company can acquire at least 3-years of market exclusivity (Bhat 2005). In this manner, the innovator-company can gain a distinguishing feature towards the generic competition. Drugs with multiple indications naturally have a higher chance of competing in different market. A crucial factor in the implementation of product-line extension strategy is the patentability of the product. Upon obtaining protection of the subsequent product innovation, it is much easier to convince a certain amount of price insensitive customer segment of the superiority of the modified drug.

The most promising, but the most difficult strategy to implement is the introduction of a follow-on product, which is either therapeutically or technologically innovative and permits better patient outcomes. The underlying rationale behind this strategy is to transfer the brand reputation as well as the patient base to the follow-on product in order to make up for the losses of sales. A key success factor for the implementation of this strategy is the interplay between two factors: price sensitivity of the market and the expected degree of improvements of drug profile. Without any significant improvements over its predecessor, it is extremely difficult to persuade the prescribers to switch to a follow-on product. The introduction of a less innovative follow-on product often receives harsh criticism from politics and funding bodies and is subject to substantial regulatory drawbacks. Another important question is whether the follow-on product should be promoted in a complementary or substitutive relationship. The sales volume following the introduction of a new product often has a cannibalizing effect on the predecessor product, but the parallel offering can avoid the risk of replacing a renowned product, which generates certain carry-over revenue without any further cost or effort. Therefore, it is advisable to grant a consolidation phase so that the full utilization of potential turnover of the previous product is not unnecessarily shortened and there is sufficient time for transition and establishment of the follow-on product on the market.

Switching the prescription drug (Rx) to an over-the-counter (OTC) drug may provide a new venue for sustained revenue, as switching is synonymous with the expansion of market segment, brought about through changes of a legal status and a business strategy. Herein, the switch means the removal of the active pharmaceutical ingredient from prescription-only status. The innovator-company can either transfer the prescription drug into an OTC status by weakening the dosages or by solely providing the drug in OTC form. The rationale underpinning the OTC-switch is the idea that the patients, who had positive experience with the branded drug, are potentially more brand-loyal in terms of product-choice. The market for OTC drug might be a more beneficial playing ground for the brand owner to exploit the value of the brand despite the expiry of patent protection. Choosing this strategic path may create new revenue opportunities by diversifying the customer base and benefit consumers with more accessible and affordable self-care solutions. However, the switching of Rx–OTC is not always practicable. The switch process is highly regulated and scientifically rigorous. For a medicine to be granted OTC status, it must demonstrate a wide safety margin and be effective, and must bear understandable labeling to ensure proper use (CHPA 2015). Furthermore, the OTC-business is characterized by low price levels and heavy advertising. Spillover effect between OTC and prescription drug, brand’s strength and marketing expertise for OTC-business constitute key success factors. At present, OTC might be a viable option in a country, where the access to appropriate medicine without prescription empowers the consumers to take control over their self-care needs.

Business model-related strategy comprises the realignment of structure and governance of transactions designed to create and capture value through an interrelated set of decision variables. Many firms do not survive in the long term despite their product innovation capabilities. The answer is clear: they failed to adjust their business models to changing environment. The literature has focused primarily on strategies related to product modification and marketing to fend off generic competition. A structured approach from the perspective of business model is so far neglected. A business model can be defined as “the logic, the data and other evidence that support a value proposition for the customer, and a viable structure of revenues and costs for the enterprise delivering that value” (Teece 2010). Visualizing it not only allows companies to get a holistic picture of the business by explaining how the firm is embedded in, and interacts with its surrounding ecosystem, but can also demonstrate the logical gaps that exist (Zott et al. 2011). The prevailing blockbuster business model that has driven the pharmaceutical industry over the last few decades is increasingly out of alignment with the technical discontinuities and changing social demands. Research focus is slowly being redirected away from blockbuster products towards therapies for stratified groups of patients, as the concept of personalized medicine promises a patient-centered clinical practice while improving the drug efficiency (Mittra and Tait 2012). Value creation in the pharmaceutical industry proceeds in a non-linear fashion with distinct inflection points. Strategic differences at firm-level may no longer be appropriate to homogenize the industry (Mittra 2007). The preferred balance between value provision and value gain depends on the distinct situational contexts within which the companies find themselves embedded. Since there is an increasing diversity in the strategic profile and innovation process, the vertically integrated value chain might transform into a service- or product platform-oriented business model by either producing diagnostic devices that aid the understanding of pharmacogenomics profile or by focusing on the discovery and pre-clinical development of active compound with a view to license it (Bigliardi et al. 2005). As the healthcare landscape is shifting towards the promise of personalized medicine and outcome-based reimbursements, a value capturing along the dimension of business model could deserve some reflection (Table 3).

**Table 3:** Overview of innovation strategies

Strategic options	Description	Exclusivity period	Source
Product-line extension	Extensions of existing drug (e.g. reformulations and combination drugs); improvement over the predecessor	Depending on the patentability of the product/3 years of market exclusivity for extensions involving clinical research	Bhat (2005), Dubey and Dubey (2009)
New indications	Finding new potential usage by extending the therapeutic indication of the drug	3 years of market exclusivity	Bhat (2005)
Introduction of follow-on products	Introduction of next-generation drug; demonstration of improved properties	Depending on the patentability of the product	Agrawal and Thakkar (1997)
Rx-to-OTC-switch	Switching a prescription drug to OTC status; expansion of the market	–	Brass (2001)
Business model innovation	Altering the firm’s core logic for creating and capturing value by specifying the value chain	–	Mittra and Tait (2012)

### Extraction strategy

The third generic strategy is to fully exploit the current market position without making additional investments in the product innovation. Two conceptually different strategic approaches can be subsumed under this category. The first strategic approach is the continuation of existing product line without any further modifications of the product offering. This strategy is aimed at securing the product turnover for as long as possible by measures related to marketing campaign or pricing strategies as well as cutting the product-related expenses before or with the expiration of the patent. In the first instance, the patent holder hopes to mitigate the declining sales volume by adjusting strategies along the dimensions of “price” and “promotion”. Brand owners normally decrease the price to compete directly with generics, but can also increase the price to reach the price-insensitive segment of the market (Grabowski and Vernon 2000; Chandon 2004). Ching (2010) confirmed that myopic firms would set higher prices to maximize short-term profit, as the incentive becomes smaller over time with uncertainty about the generic quality slowly resolving. However, these only have a marginal effect of delaying the decline in sales. Sooner or later, these strategies need to be abandoned, as the increasing costs of the measures would not justify the additional revenues generated through promotional campaign in the long term. Even though, the price is rather a simple and easy to customize dimension, there is still limited price sensitivity on the side of consumers (patients) and the decision makers (prescriber), because it is normally the statutory health insurance, which ultimately bears the costs and determines the price structure. Nevertheless, the pace and extent of the decline in sales is closely related to the existing price differential. It is therefore important for the brand owners to make hypotheses about the entry-level price of the generic drugs, because the care providers take account of the price ratio involved for the patient when writing the prescriptions for medication or specialized care. A reactive or retroactive price adjustment allows generic suppliers to establish themselves in the market at an early stage. Since the recapturing of the lost market share is complex, brand owners have to deal with the price adjustment several months prior to the patent expiry as a pro-active contribution to the marketing strategy. In addition, a price reduction may adversely affect the international price grid, as it can trigger parallel export to countries, where the drug is still protected by a patent (Raasch 2009). Consequently, it is critical to anticipate the favorable price ratio for the implementation of price reduction strategy. The brand owner must take into account that generic providers are also willing to cut down the prices in order to maintain an adequate level of price gap. For example, the extraordinary success of the drug Neurontin after its patent expiration lies in the fact that the generic product was only about 15 % lower priced than its original—a difference, for which the prescribers can waive the use of generics. On the other side, the generic version of Pfizer’s Lipitor Atorvastatin Hexal^®^ offered a price advantage of up to 85 % upon its launching. Hence, the company must be aware of its own limit to effectively participate in a price war against the generic producers. Moreover, the patent holder can intentionally defer the marketing expenses and reduces expenditures on internal and external sales force dedicated to the blockbuster drug. The goal here is to let the product phase out with the loss of market exclusivity. Thus, no resources are being poured into combating the generic competitors, as the chances of success appear to be low. The product still can generate a certain turnover with “loyal prescribers”, who do not shift their patients to low cost alternatives. In such cases, the company does not wish to strive for a strong presence in the product line concerned and has to accept that sooner or later it will confronted with a substantial sales drop. The second strategic approach under this category is the selling or licensing a patent to generic suppliers before the patent expiry. Instead of a controlled, strategic withdrawal from the product line, the selling or out-licensing of compounds (existing patent right and trademark) to other entities may turn out to be more appropriate. Licensing might benefit the patent holder through externalities from standards creation or from access to the licensee’s complementary technologies (Somaya 2003). Moreover, it might be attractive to out-license the drug to another company to explore for alternate indications. Chong and Sullivan (2007) argued that “repurposing” of old drug has proved successful in bringing new therapies and there are approximately 8850 unique drugs worth of screening. The detailed specifications of such licensing agreements are diverse, whereby it is necessary to make the fundamental decision as to whether the innovator-company wants to participate in the product success by covering a part of the risk or whether it prefers to transfer the right against the one-off payment. For smaller companies with a scarcity of available products, the residual potential of the blockbuster drug is an important incentive for active commercialization. An authorized generic entry is profitable, if the licensing income exceeds the losses resulting from the generic entry. The first-mover advantage often grants a sustainable competitive edge over subsequent entrants (Hollis 2002). Hence, the pricing process must be critically reviewed, especially as early entries can be accompanied by the aggressive development of market prices and heavy losses of sales of branded drugs. Reaching an early entry agreement is also known to be critically involved in affecting the attractiveness of subsequent generic entry and decreasing the intensity of off-patent competition (Table 4).

**Table 4:** Overview of extraction strategies

Strategic options	Description	Effect	Source
Continuation of existing product line	Leverage promotional campaign and pricing strategies to maximize the potential turnover	Short-term	Kvesic (2008), Raasch (2009)
Differentiation	Competitive advantage through brand recognition; strong brand image	Mid-term	Agrawal and Thakkar (1997)
Exit strategy	‘Milking’ of the product; letting the product slowly phase out	Short-term	Chandon (2004)
Licensing	Licensing or selling of the exclusive rights to generic manufacturers	Short-term	Glasgow (2001)

### Adaption strategy

The last generic strategic pathway is to introduce branded generics by establishing itself as an active player in the generic market. Two conceptually similar strategic alternatives fall into this category: (1) developing a low-cost alternative in the form of wholly owned subsidiary focusing on generic drugs, which operates separately outside the main organization (Gilbert and Strebel 1987); (2) offering of a generic through the innovator-company itself (Agrawal and Thakkar 1997). The basic principle of ‘adaption strategy’ is therefore to retain existing customer base through an active entry into the business of generic drug and to capture a share of the generic profits. In small markets, branded generics may deter generic entry, as they have a manufacturing advantage. The underlying rationale behind the launch of generic by subsidiaries is to leverage synergies and to diversify the product portfolio by having a strong presence in different key markets. In the past, the attempt to gather the innovator-company and generic suppliers under the same roof ended up with failure. This comes from the circumstance that the generic business requires short reporting and decision-making channels as well as the competence in market research and admission process rather than in R&D. Exploring the fit between the internal organization and its business model has a significant impact on firm performance (White 1986). But today, many established brands have their own generic subsidiary (e.g. Sandoz from Novartis Group) and intentionally separate branded and generic divisions in order to provide an enhanced ability to respond to market dynamics.

The launch of a “second brand” (a so-called fighter brand) with an additional branded product sold at a discounted price under its own corporate brand is frequently discussed as a potentially promising new strategic option, but has been rarely implemented because of its credibility for the external world. Raasch (2008) confirmed that the rationale behind the launch of a fighter brand is “a segmentation of demand according to price sensitivity, maintaining a higher profit margin on the units sold […] but not entirely losing more price-sensitive prescribers”. Similarly, a company considering the launch of a fighter brand can receive following competitive advantages: (1) brand popularity and reputation as the producer of the proven drug will likely benefit the new brand; (2) the brand owner can take advantages of already existing manufacturing infrastructure to profit from learning curve-based cost advantages (Raasch 2008). A downside would be the fact that the commercialization of low-priced products is often incompatible with the corporate image as well as culture and the business model of the research-driven company. Also it would generate a cannibalizing effect on the original drug. The critical issue for implementing this strategy is whether the discounted drug should substitute the high-priced branded drug or both are expected to complement one another. In both cases, it opens up the possibility of securing a part of the market falling to generics, thereby exploiting the experience-related advantages in the synthesis and packaging of the drug and contributes to the coordinated utilization of existing production capacities. However, the first approach can gain a substantial market share, only if the subsidiary brings its generic to the market in the course of early entry before other generic competitors, thereby realizing the first-mover advantage. Therefore, it is imperative to consider the likely development of the price corridor after the generic entry. A proactive pricing behavior and an aggressive marketing communication towards the prescribers would positively contribute to the success of launching secondary brand product (Table 5).

**Table 5:** Overview of adaption strategies

Strategic options	Description	Effect	Source
Branded generics (1)	Offering generic through subsidiary	“First mover advantage”; serving different markets	Gilbert and Strebel (1987)
Branded generics (2)	Offering generic through brand owner	Serving different customer segments	Raasch (2008)

**Fig. 3:**
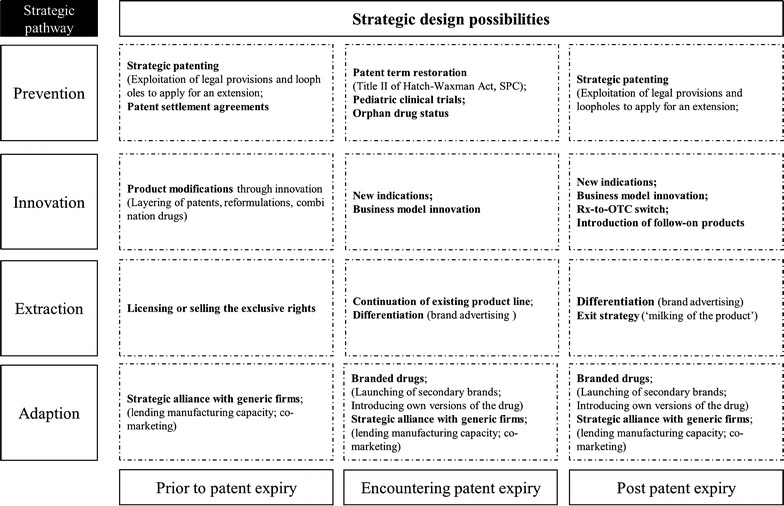
Framework of the strategic and tactical design possibilities in the pharmaceutical industry (*Source*: own figure)

To prolong the effective patent life for the drug beyond the expiry date of the patent, companies can take various pathways defined by the different stages of the product lifecycle (Chandon 2004). Determining which pathway to pursue depends on company’s capabilities, priorities and the chosen time period. Not all options are available at a given time. There are distinct phases of product lifecycle of the blockbuster drug to consider, if one wishes to extend its period during which they can shield from generic competition. A healthy mix of innovative and legislative efforts to promote pharmaceutical innovation is the key ingredient in enabling the company to generate a platform for future growth and to preserve market dominance after patent expiration. An important determinant for success within the framework of innovation strategy is not only to innovate, but also to successfully patent the inventive output. Therefore, scientist and patent lawyers need to communicate and cooperate effectively in order to identify a common denominator. Pharmaceutical scientists are in need of having a better understanding of the patent fundamentals, as they often do not realize that an improvement they made could be a patentable subject matter. A lack of interaction between these two groups and understanding of each culture may lead to missed opportunities in terms of competitiveness that are both impeding and costly to the firm development (Fig. 3).

A framework for designing various strategic possibilities been compiled. Taken together, it creates a large strategic diversity for the incumbent company and adds to the complexity of designing ideal patent expiration strategies. For some companies, the remaining time until the patent expiry may not be sufficient or the necessary investment may be too high to allow appropriate adjustments to be made in time or pursuing a certain strategy may appear to be not in accordance with the management philosophy. The dynamics of drug prices and the competition by generic drugs vary also significantly across cultures (Magazzini et al. 2004). The profitability of early entry (“authorized generics”) or launching of “fighter brand” depends significantly on the accuracy of the firm’s forecasting capabilities. Replacement of branded product by follow-on product and the product modification strategies are generally employed through secondary patenting. Innovation strategy requires a sufficient lead time, a multidisciplinary approach and a well-coordinated program for efficient processing. Therefore, the prevention and innovation strategy need to be performed both in sequence and in parallel. The interplay among various strategies should be constantly reassessed in order to offer the best competitive defense. Translating a strategic orientation into an actual advantage requires further choices about how much investment to make and what approach should be taken. Thus, a firm must identify its current position and requires a careful assessment of one’s capabilities and limits. In total, a greater emphasis has been placed on employing combined lifecycle management strategies by research-driven pharmaceutical companies, as generic competition becomes stronger and more sophisticated. The case of patent cliff should be perceived as “social laboratory” predicting future trends in industrial dynamics and competition in the pharmaceutical industry and as such it is a model for outcome-based payments for some aspects. Hence, it is important that societal consensus is maintained for solutions in this area, and multi-stakeholder platforms (including policy makers, payers and industry representatives) should work on improving communication and awareness of the issue by collaboration and by sharing expertise.

## Conclusions

The patent expiry is one of the most important events within the life cycle of pharmaceutical products. In view of diminishing product pipeline, a growing significance is attached to the active marketing of the existing products—even beyond the expiry of patent protection. As it is becoming increasingly difficult for major pharmaceutical companies to continuously develop new blockbuster drugs on a regular basis, companies can consider developing their own generic versions of blockbuster drug to minimize the loss to their brand or offer promotional consumer discounts by reinventing themselves as a more customer-centric organization. The development of an extension strategy requires an intensive interplay among following components. (1) A thorough understanding of the business environment and anticipation of possible shifts in the pattern of industry dynamics; (2) devising strategies that deliver a competitive advantage for perceived value (e.g. product dimension) or delivered cost (e.g. price dimension); (3) promoting the collaboration between scientists, attorneys and marketing manager in order to make maximum use of a global view of product lifecycle management; (4) a combination of product modification, promotional and pricing strategies. Most strategies involve interdisciplinary knowledge on marketing, intellectual property rights and technologies. Future research in these areas may benefit from the extensive literature on technology and innovation marketing in other industries. Moreover, the discussed strategies are mostly based on non-biologic drugs. The approval processes for biosimilars (generics of biologics) are subject to different technical and legal procedures, which are more complex in general. A comprehensive analysis dedicated to biosimilar market is needed to address these questions in a significant manner. In addition, little attention has been paid to how an optimal pricing, promotion and divestiture process might look like and when to implement a mixed-strategy and when not. Thus, firm’s knowledge, reputational and relational assets should be carefully managed in a coherent manner to aid the decision-making process.

An open question is how aggressively brand owners are willing to support the drug with investments aimed at differentiating it from generic competitors. Branded pharmaceuticals have certain attributes that lead to different patterns of losing market share after they go off patent. A rapid erosion of market share is more likely to occur, if drugs are easier to replicate or are less subject to managed care control (Tuttle et al. 2004). Thus, the challenge lies not only in evaluating the potential, but also in arriving at a reliable forecast. Given the wide range of possible outcomes upon facing generic entry, understanding the specific attributes and their relationship to the brand’s intrinsic potential are critical to make the right strategic decisions about post-patent expiry and to sustain the franchise. Hence, the innovator-company needs to treat patent-related (i.e. regulatory) and factual strategies both as weapon and a shield to outpace the generic competition. The product-related strategies need to be supported by a consistent pricing and promotional strategy. Within a firm, several departments are involved in making decisions that related to strategic balancing of market exclusivity, but not every department has the same knowledge or awareness of the strategic possibilities of prolonging the product life (Dolfsma 2011). The provided scheme can serve as a fertile ground for assessing the suitability of the measures chosen to achieve the desired objective, broadening company’s perspectives and consensus-building for addressing the issues arising from the patent expiry. Patent expiration is not the end of the product life, but with a proper balancing, it can be a second beginning.

## Footnotes

1 The innovator-company, research-driven company, brand owner and incumbent are synonymous.

2 Pharmaceutical products are usually protected by a large number of product and process patents. Instead of facing single patent expiration, there are several successive ones. In most cases, there is one main patent protecting the active compound and several secondary patents protecting aspects other than main patent. Upon patent expiry, if generic companies cannot find ways to bypass a patent protecting the supporting aspects, it can act as a competition limiting factor.

3 Chemical compounds often appear in different isomeric forms (i.e. having the same chemical structure, but differs in spatial arrangement), such as *cis*/*trans*-isomers or enantiomers. Isomers may demonstrate different biological activity.

4 Paragraph IV Certification contains the claim of a generic firm that the patent of brand owner is not infringed or is invalid.
